# Selective use of pancreatic duct occlusion during pancreaticoduodenectomy in patients with a small-size duct and atrophic parenchyma in the distal pancreas: A retrospective study

**DOI:** 10.3389/fsurg.2022.968897

**Published:** 2023-01-06

**Authors:** Guangbin Chen, Jie Yin, Qun Chen, Jishu Wei, Kai Zhang, Lingdong Meng, Yichao Lu, Pengfei Wu, Baobao Cai, Zipeng Lu, Yi Miao, Kuirong Jiang

**Affiliations:** ^1^ Pancreas Center and Department of General Surgery, The First Affiliated Hospital of Nanjing Medical University, Nanjing, China; ^2^ Pancreas Institute, Nanjing Medical University, Nanjing, China; ^3^ Department of Hepatobiliary Surgery, Wuhu Hospital Affiliated to East China Normal University, Wuhu, China; ^4^ Pancreas Center, The Affiliated BenQ Hospital of Nanjing Medical University, Nanjing, China

**Keywords:** pancreaticoduodenectomy, pancreatic duct occlusion, pancreatic fistula, pancreatic atrophy, pancreas

## Abstract

**Background:**

Despite the advancements in surgical techniques, postoperative pancreatic fistula (POPF) remains a potentially life-threatening complication of pancreaticoduodenectomy (PD). Pancreatic duct occlusion (PDO) without anastomosis has also been proposed to alleviate the clinical consequences of POPF in selected patients after PD.

**Objectives:**

To assess the safety and effectiveness of PDO with mechanical closure after PD in patients with an atrophic pancreatic body-tail and a small pancreatic duct.

**Methods:**

We retrospectively identified two female and two male patients from April 2019 to October 2020 through preoperative computed tomography of the abdomen. Among them, three patients underwent PDO with mechanical closure after PD, and one underwent PDO after pylorus-preserving PD. In addition, patients' medical records and medium-and long-term follow-up data were analyzed.

**Results:**

Postoperative histological examination revealed a solid pseudopapillary tumor in two patients, pancreatic ductal adenocarcinoma in one patient, and chronic pancreatitis with pancreatic duct stones in one patient. However, none of the patients developed biochemical or clinically relevant POPF, with no postpancreatectomy hemorrhage, biliary leakage, delayed gastric emptying, intra-abdominal abscess, or chyle leakage. Among the four patients, three developed new-onset diabetes mellitus, and one had impaired glucose tolerance. Furthermore, three patients received pancreatic enzyme supplementation at a dose of 90,000 Ph. Eur. units/d, and one was prescribed a higher dose of 120,000 Ph. Eur. units/d.

**Conclusions:**

PDO with mechanical closure is an alternative approach for patients with an atrophic pancreatic body-tail and a small pancreatic duct after PD. Therefore, further evidence should evaluate the potential benefits of selective PDO in these patients.

## Introduction

Pancreaticoduodenectomy (PD) is one of the most complex and challenging procedures in abdominal surgery. Postoperative pancreatic fistula (POPF) is a major severe complication of PD, with a reported incidence of 10%–15% (1–3). POPF is a lethal complication that can significantly prolong hospital stays and increase healthcare costs and postoperative mortality (4, 5). The design and surgical technique for pancreatic-enteric anastomosis are significant determinants of POPF; however, no preferred re-establishing procedure has been demonstrated to be superior to others (5–7).

Pancreatic duct occlusion (PDO) without pancreatic anastomosis has been explored as an alternative to mitigate pancreatic fistulas following PD. The rationale behind this surgical design is that when a pure pancreatic fistula forms, it is not triggered by biliary and/or enteric juices, thereby reducing the risk of serious pancreatic fistula consequences. Occluding the pancreatic duct has been reportedly associated with a high rate of biochemical leakage, which is self-limiting, with no deviation from the clinical pathway (8, 9). Postoperative pancreatic exocrine and endocrine functions are similarly preserved in patients who have undergone PDO compared with those who have undergone pancreatic anastomosis (10). During pancreatic anastomosis in patients with a small pancreatic duct in PD, identifying the duct and securing the anastomosis remain technical challenges. Therefore, we hypothesized that PDO is a good alternative to pancreaticojejunostomy in this clinical scenario. Since 2019, we have selectively used PDO with a linear stapler in PD for patients with a small duct and atrophic parenchyma in the distal pancreas. This study aimed to evaluate the safety and efficacy of this technique in a selected patient cohort.

## Materials and methods

### Study design and population

We retrospectively identified four patients (cases 1–4) with pancreatic atrophy and a small pancreatic duct who underwent PDO by the same team of surgeons after PD at our center, which is a high-volume institution for pancreatic surgery in China, from April 2019 to October 2020 (11, 12).

Patients underwent either open PD or pylorus-preserving PD (PPPD) for any disease with ductal occlusions of the pancreatic duct of the distal remnant (without re-establishing pancreaticojejunal anastomosis [PJA]). In all cases, the pancreatic stump was closed using a linear stapler device (Echelon Flex EC60A with a 2.5 mm staple load; Ethicon-Endo Surgery™). Three abdominal silicon drainages with gravity suction were placed (two proximal to the pancreatic remnant and one posterior to the hepaticojejunostomy) to ensure effective surveillance and drainage of any possible POPF. Passive drainage with gravity was applied after surgery, and amylase level in the drainage fluid was routinely measured on postoperative days 1, 3, and 5 and on additional days when needed. Finally, drains were usually removed on postoperative day 7 when a fistula was not observed.

### Definitions

Pancreatic atrophy was defined as a pancreatic body width of < 10 mm on preoperative computed tomography (CT) (13). Classification of the pancreatic duct size and texture, which predicts POPF, was based on the consensus of the International Study Group of Pancreatic Surgery (ISGPS) (14). POPF, postpancreatectomy hemorrhage (PPH), and delayed gastric emptying (DGE) were defined and graded distinctively according to ISGPS criteria (15–18). In addition, the diagnosis of biliary leakage (BL) and diabetes mellitus (DM) was performed following the International Study Group of Liver Surgery recommendations (19) and the American Diabetes Association recommendations (20), respectively.

### Data collection and analysis

Patient's medical records, including clinical history, preoperative investigations, intraoperative data (texture of the pancreatic remnant and main pancreatic duct size), postoperative complications, postoperative hospital stays, and postoperative pancreatic endocrine and exocrine functions, were prospectively collected and retrospectively analyzed. Morbidity and mortality were evaluated within 30 days of surgery or during hospitalization. Medium-and long-term follow-up data were acquired from the same medical database at the pancreatic center. General routine examinations, including hematologic and biochemical examinations, as well as abdominal CT, were performed postoperatively in all patients. Follow-up evaluations were conducted *via* telephone interviews or outpatient service visits. The endocrine pancreatic function was indirectly evaluated by measuring the postoperative fasting blood glucose (FBG) level and performing an oral glucose tolerance test (OGTT). Conversely, the exocrine pancreatic function was evaluated based on symptoms of steatorrhea and the dosage of pancreatic enzyme supplementation.

### Ethics statements

The study protocol was reviewed and approved by the Ethics Committee of the First Affiliated Hospital of Nanjing Medical University (Jiangsu Provincial People's Hospital). All patients provided written informed consent before participation in this study.

## Results

**Figure 1:**
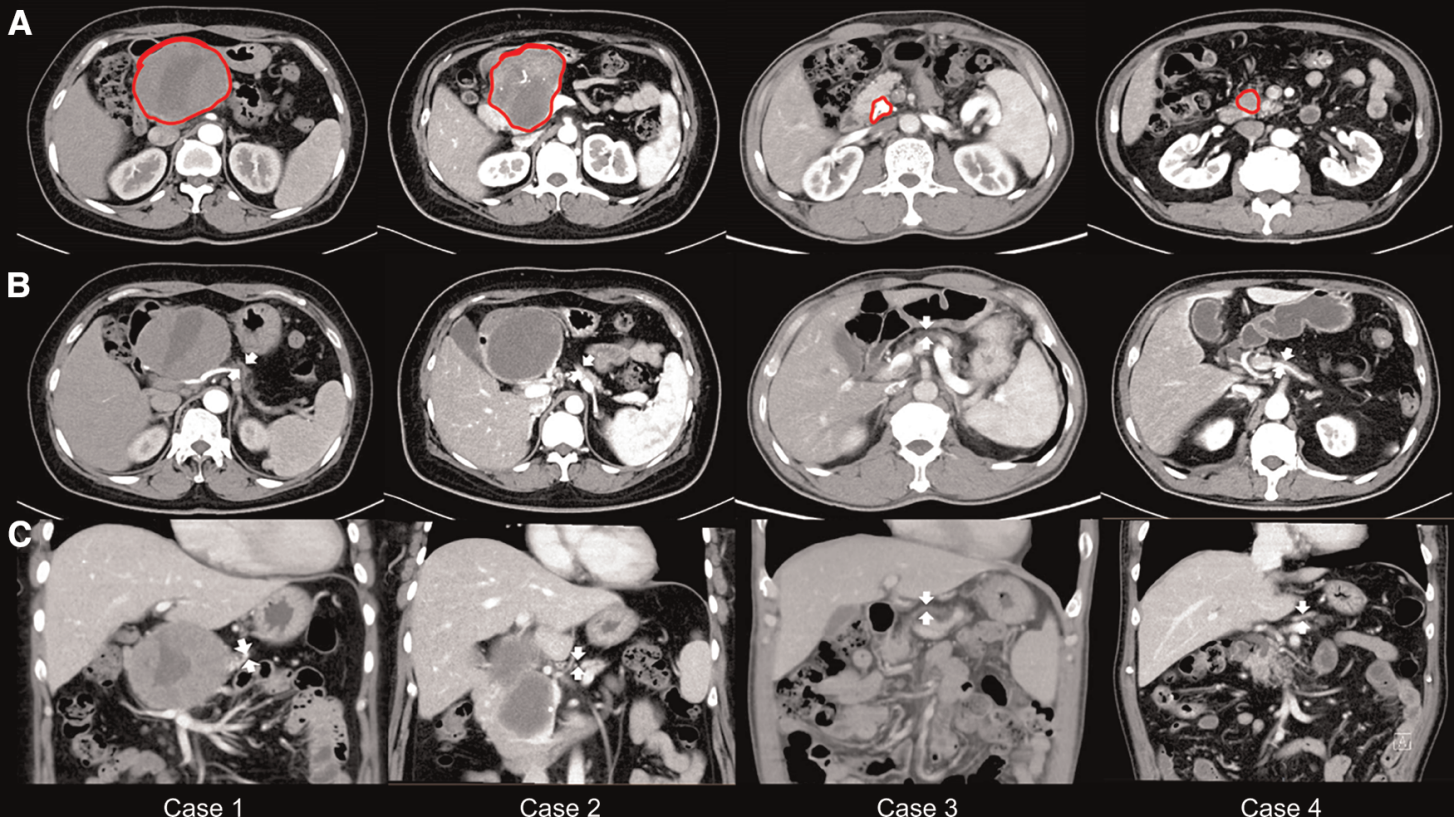
Preoperative abdominal computed tomography scans. (**A**) The tumor or stone is marked in red. (**B**) The width of the pancreatic bodies is < 10 mm, with arrows highlighting the atrophy of the pancreatic bodies. (**C**) The thickness of the pancreatic bodies is < 10 mm in the image, with arrows indicating the atrophy of the pancreatic bodies.

This study included two female and two male patients with atrophic glands and small ducts in the left pancreas who underwent PDO without pancreaticojejunostomy during PD. These patients (age range, 40–63 years) were diagnosed with a pancreatic head lesion with atrophy in the distal part preoperatively on an abdominal contrast-enhanced CT, and the width of their pancreatic body ranged from 7 to 9 mm (Table 1; Figure 1). The physical status of all patients was categorized as American Society of Anesthesiologists grade II. None of the patients had a history of DM, and preoperative FBG levels were between 5.39 and 5.99 mmol/L (Table 1). In addition, none of the patients complained of symptoms of exocrine pancreatic insufficiency, such as steatorrhea or oral pancreatic enzyme supplements before surgery.

**Table 1:** Baseline patient data.

	Case 1	Case 2	Case 3	Case 4
Sex	F	F	M	M
Age, year	55	40	57	63
BMI, kg/m^2^	24.8	29.1	22	24.8
ASA grade	II	II	II	II
Comorbidity	None	None	CI, 10 year	HT, 20 year
Preop. endocrine function				
DM history	None	None	None	None
Preop. FBG level, mmol/l	5.62	5.77	5.39	5.99
Preop. exocrine function				
Steatorrhea	None	None	None	None
Enzyme supplement, mg	None	None	None	None
Pancreatic duct size*, mm	1.3	1.5	1.2	Unmeasurable
Pancreatic body width*, mm	9	8	7	8
Parenchyma thickness at the transection line*, mm	7	7	8	6

* Measurements were obtained from preoperative computed tomography scans.

ASA, american society of anesthesiologists, DM, diabetes mellitus, CI, cerebral infarction, HT, hypertension, preop, preoperative, FBG, fasting blood glucose, BMI, body mass index.

In total, one and three patients underwent PPPD and PD, respectively. The operative duration and intraoperative blood loss ranged from 252 to 400 min and 200 to 400 ml, respectively. Based on the ISGPS POPF risk stratification, all patients were classified as type B with a non-soft pancreatic texture and a small duct (Table 2). In addition, the postoperative histological examination revealed a solid pseudopapillary tumor in two patients, pancreatic ductal adenocarcinoma in one patient, and chronic pancreatitis with pancreatic duct stones in one patient (Table 2).

**Table 2:** Intraoperative data and postoperative pathology findings.

	Case 1	Case 2	Case 3	Case 4
Procedure	PD	PPPD	PD	PD
Texture of the pancreatic stump (not soft/soft)	Not soft	Not soft	Not soft	Not soft
ISGPS POPF risk classification*	B (6.2%**)	B (6.2%)	B (6.2%)	B (6.2%)
Operative time, min	300	400	252	275
Estimated blood loss, ml	200	300	300	400
Postoperative pathology findings	SPT	SPT	CP	PDAC

PD, pancreaticoduodenectomy, PPPD, pylorus-preserving pancreaticoduodenectomy, SPT, solid pseudopapillary tumor, CP, chronic pancreatitis, PDAC, pancreatic ductal adenocarcinoma, ISGPS, international study group of pancreatic surgery.

* This classification was proposed by the ISGPS in 2021 (14).

** The incidence of pancreatic fistula in class B is 6.2%.

However, none of the patients showed biochemical leakage or clinically relevant POPF (grades B and C). No cases of PPH, DGE, BL, intra-abdominal abscess, intra-abdominal fluid collection, or wound complications were observed (Table 3). None of the patients required an interventional procedure or had a 90-day readmission, and the 90-day mortality was not recorded. Furthermore, the mean postoperative hospital stay was 11 days (range, 9–16 days) (Table 3).

**Table 3:** Postoperative course and complications.

	Case 1	Case 2	Case 3	Case 4
Complication				
Biochemical leakage	None	None	None	None
POPF (grades B+C)	None	None	None	None
DGE	None	None	None	None
PPH	None	None	None	None
BL	None	None	None	None
Intra-abdominal abscess	None	None	None	None
Intra-abdominal fluid collection	None	None	None	None
Chyle leakage	None	None	None	None
Wound complication	None	None	None	None
Reoperation	None	None	None	None
Postoperative hospital stay, d	9	16	12	10
90-day readmission	None	None	None	None
90-day mortality	None	None	None	None

POPF, postoperative pancreatic fistula, DGE, delayed gastric emptying, PPH, postpancreatectomy hemorrhage, BL, biliary leakage.

**Figure 2:**
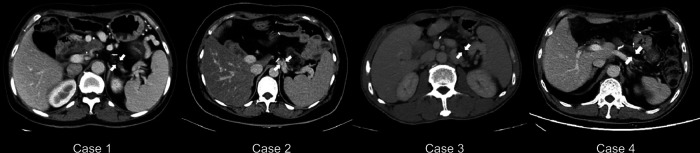
Abdominal CT scans during the follow-up studies. Abdominal CT reveals that the distal pancreas is intact in all cases (indicated by arrows). CT = computed tomography.

**Figure 3:**
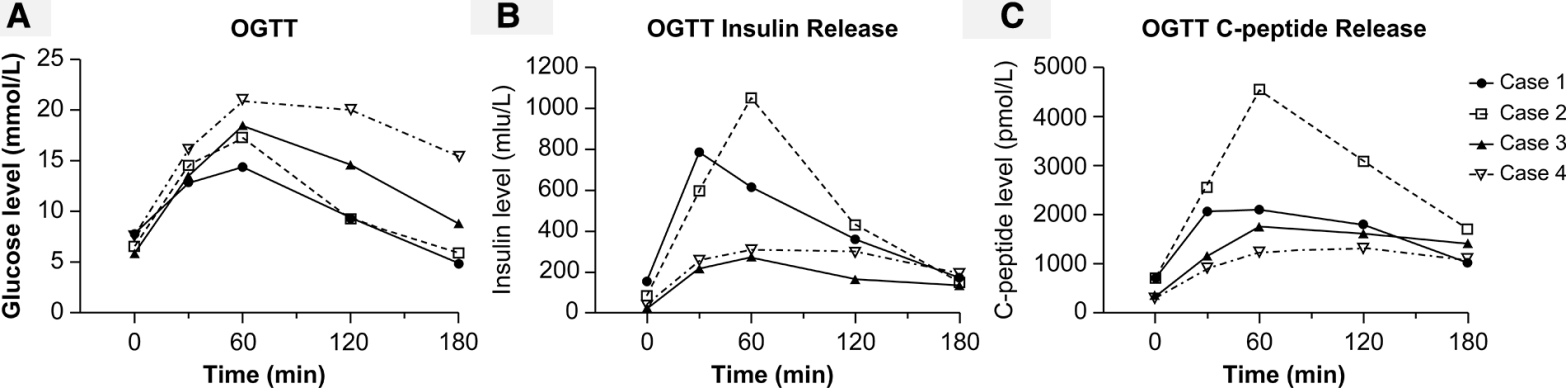
Results of pancreatic endocrine function, including the 75 g OGTT during the follow-up studies. (**A**) OGTT curve; (**B**) OGTT insulin-release curve; and (**C**) OGTT C-peptide release curve. OGTT = oral glucose tolerance test.

All four patients survived over an average follow-up period of 13 months (range, 11–26 months. The patients underwent abdominal CT during the follow-up assessments (Figure 2), where imaging showed that the distal pancreatic remnant was well preserved because of sufficient blood supply, with no indications of inflammation or further atrophy (Figure 2). Patients' median short-term postoperative FBG level was 6.69 mmol/L (range, 6.32–7.5 mmol/L) (Table 4). Therefore, to evaluate the median-term change in glucose metabolic status after the procedure, all four patients underwent a 75 g oral OGTT at a median time of 13 months postoperatively (range, 11–26 months) (Table 4). Three patients developed new-onset DM (3/4), whereas one (case 2) had impaired glucose tolerance (IGT). Insulin and C-peptide levels were also measured to further evaluate the pancreatic endocrine function (Figures 3A–C). The insulin levels ranged from 21.4 to 149.6 mIU/L and 271.8 to 1058 mIU/L for the base and peak values, respectively; the base and peak values for C-peptide levels ranged from 286.4 to 707.1 pmol/L and 1234 to 4568 pmol/L, respectively. In addition, the peak times were 30 to 120 min. Because of the patient's diabetic status, cases 1, 3, and 4 had flat release curves. However, case 2 showed a release curve with a delayed peak (Table 4; Figures 3B–C). All patients received oral pancreatic enzyme supplementation at a dosage of 90,000 Ph. Eur. units/d after discharge. During the follow-up, only one patient (case 3) reported steatorrhea after consuming fatty food, which was alleviated after adjusting the pancreatic enzyme dosage to 120,000 Ph. Eur. units/d (Table 4).

**Table 4:** Postoperative pancreatic endocrine and exocrine function results.

	Case 1	Case 2	Case 3	Case 4
Follow-up time, months	26	14	12	11
Endocrine function results				
Postop. FBG level, mmol/L	7.5	6.52	6.32	6.86
FU OGTT at 0 h, mmol/L	7.81	6.55	5.92	7.6
FU OGTT at 2 h, mmol/L	9.39	9.26	14.62	20.03
FU insulin usage, U/d	17	None	15	6
FU antidiabetic drug usage, mg/d	None	None	None	Repaglinide, 1.0
Status	DM	IGT	DM	DM
FU exocrine function results				
Steatorrhea	None	None	None	None
Enzyme supplement, Ph. Eur. units/d	90,000	90,000	120,000	90,000

Preop, preoperative, postop, postoperative, FBG, fasting blood glucose, FU, follow-up, OGTT, oral glucose tolerance test, DM, diabetes mellitus, IGT, impaired glucose tolerance.

## Discussion

With the recent advances in medical technology and perioperative management, the mortality rate after PD has decreased to < 5% (21–23); however, the morbidity rate remains high at 30%–50% (24–26). A pancreatic fistula is the most common cause of morbidity and mortality after pancreatic anastomosis (27, 28). Furthermore, the activation of pancreatic enzymes from the distal pancreas by intestinal and/or biliary juices is the major cause of complications related to POPF, leading to erosion of the anastomosis, leakage of the biliary-enteric anastomosis, intra-abdominal abscesses, severe sepsis, hemorrhage, and DGE (1, 9, 27). However, the consensus risk factors associated with POPF include a small pancreatic duct (≤ 3 mm) and soft pancreatic texture (3, 4, 29, 30), with a soft pancreatic texture reflecting active exocrine function and a small-sized pancreatic duct requiring a more precise anastomosis of the pancreatic duct (5, 9, 14). Therefore, various strategies, including preoperative, intraoperative, and postoperative interventions and management, have been attempted to prevent the development of POPF, particularly surgical procedures (5, 27, 31–35).

According to the 2017 ISGPS statement (7), no universal standard modality is currently available for reconstruction after pancreatic surgery to avoid clinically relevant POPF. Nonetheless, surgeons dedicated to the pancreas have attempted to overcome these difficulties and make individualized decisions based on the clinical characteristics of patients and the pancreas (e.g., metabolic comorbidities, pancreatic duct diameter, pancreatic fibrosis, and the degree of tissue inflammation).

PDO/closure without pancreatic-enteric anastomosis after PD has been proposed to mitigate clinically relevant POPF, with the pancreatic remnant injected with chemical substances, stapled, or sutured (8–10, 32). This approach is technically simple, relatively safe, and less labor-intensive than pancreatic anastomosis. Moreover, occluding the pancreatic duct can prevent pancreatic enzyme activation by enteric and/or biliary juices in the case of POPF. A prospective, non-randomized clinical study in 2019 revealed that the early postoperative outcome of selected patients at high risk of POPF undergoing PDO with glue injection is equivalent to that of patients at low risk of POPF undergoing PJA (32). Although Mauriello et al. (8) used a linear stapler and Alfieri et al. (9) injected glue, they both reported that the occlusion of the pancreatic duct was related to a high rate of grade A fistulas (redefined as “biochemical leak” in 2017 [15]), which were considered self-limiting with no deviation from the conventional postoperative clinical pathway. Hemorrhage is another major complication of PD for artery skeletonization after curative lymph node dissection, exposing activated pancreatic enzymes and subsequent eroding (9). Regarding hemorrhage, pancreatic anastomosis leakage after PDO could be less threatening than PJA. Furthermore, endocrine and exocrine pancreatic insufficiency are other concerns for surgeons after surgery. Postoperative pancreatic exocrine insufficiency is common in patients with pancreatic cancer, and 74% of those undergoing PD with PJA require enzyme supplementation (36). Alfieri et al. (10) conducted long-term follow-ups objectively and subjectively after PD and reported no significant difference in postoperative pancreatic exocrine and endocrine functionality between PDO and PJA. Despite a higher frequency of objective exocrine insufficiency in the PDO group, the need for postoperative substitutive enzymes did not increase (10).

To date, several reports have focused on the differences in POPF and pancreatic functional outcomes between PDO and PJA after PD, with few studies on patients with pancreatic atrophy and a small pancreatic duct undergoing PD. Therefore, it is difficult to effectively perform pancreaticojejunostomy in a patient with a narrow pancreatic duct, which may lead to the risk of severe POPF. This study's innovative characteristics depend on surgical decision-making, which considers the type of anastomotic reconstruction and fibrosis/atrophy of the pancreatic remnant with a small pancreatic duct, as well as the recommendations for selected patients undergoing PD who are eligible for PDO without anastomosis. Since a pancreatic head tumor or pancreatic duct stone obstruction of the main duct induces atrophy of the body and tail of the pancreas (37–39), PDO can be practically considered a typical model of exocrine pancreatic insufficiency due to atrophy of the pancreatic remnant (10). In this study, four selected patients with an atrophic pancreatic body tail and a small pancreatic duct underwent PDO without anastomosis after PD/PPPD. Three patients developed new-onset DM, and one developed IGT. All four patients received pancreatic enzyme supplementation postoperatively. Furthermore, pancreatic insufficiency after surgery is expected, considering fibrosis and/or atrophy of the distal pancreas and loss of partial function preoperatively.

This study was limited due to its retrospective, non-randomized nature and involved a few patients with an atrophic pancreatic body tail and a small pancreatic duct. Nevertheless, based on this study and an extensive literature review, PDO is an alternative procedure for patients with an atrophic pancreatic body-tail and a small pancreatic duct in PD. Furthermore, PDO after PD may be considered in selected “high-risk” patients, particularly those with a soft pancreas and small pancreatic duct, to prevent and reduce POPF-related complications (8, 9, 32). In addition, PDO remains an option to manage the pancreatic remnant and prevent the completion of pancreatectomy in “difficult circumstances,” such as severe POPF requiring relaparotomy (40, 41).

## Conclusions

Although a larger sample size is required to confirm our results, this study suggests that PDO with mechanical closure is a safe, easy-to-perform preferred procedure to manage the pancreatic stump during PD in patients with an atrophic pancreatic body tail and a small-sized duct. However, the long-term function of the remnant pancreas after this procedure warrants further evaluation.

## Data Availability

The original contributions presented in the study are included in the article/Supplementary Material, further inquiries can be directed to the corresponding author/s.
